# Closed-Loop Optogenetic Control in a Microplate Reader

**DOI:** 10.1021/acssynbio.6c00003

**Published:** 2026-05-25

**Authors:** Hari R. Namboothiri, Krishna Pochana, Bhavya Jaiswal, Azita Emami, Chelsea Y. Hu

**Affiliations:** † Department of Chemical Engineering, 14736 Texas A&M University, College Station, Texas 77843, United States; ‡ Division of Engineering and Applied Science, 6469 California Institute of Technology, Pasadena, California 91125, United States

**Keywords:** LEMOS, optogenetic feedback control, growth-aware gene expression dynamics, cybergenetics

## Abstract

Optogenetics integrates living cells and electronics into powerful cell–silicon systems, but prototyping their dynamics remains challenging. Current tools either require robotic liquid transfers into flow cytometers or rely on custom sensors with a narrow dynamic range that limits controller performance. Additionally, current successful optogenetic feedback controllers only operate in chemostats or microfluidic devices that enforce constant growth, because models for growth-aware controller design in batch culture are lacking. Here, we present LEMOS, a low-cost LED-embedded microplate that runs inside a commercial microplate reader. Coupled with a growth-aware multiscale model of gene expression for controller tuning, this platform enables rapid design-build-test-learn cycles for cell-silicon systems. We demonstrate closed-loop set point tracking of gene expression in batch cultures within a standard microplate reader and show how growth dynamics complicate controller selection and tuning. Together, this platform reduces setup overhead and speeds up iteration, enabling accurate real-time optogenetic feedback control.

## Graphical abstract

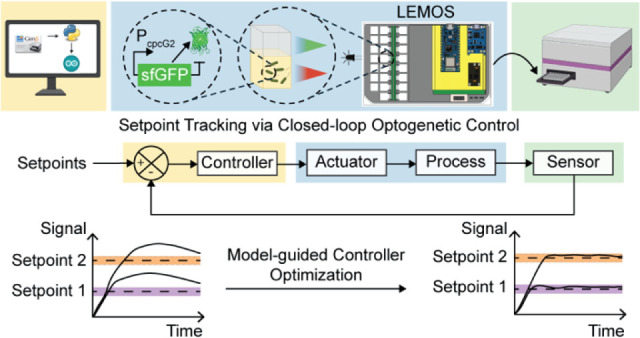

## Introduction

Living cells, shaped by evolution, sense a wide diversity of molecules and convert these signals into coordinated gene expression and the synthesis of complex organic compounds. Yet, cellular responses are slow, noisy, and context-dependent, which makes precise regulation difficult. Electronic systems have limited direct access to chemicals but excel at high-speed signal transduction, computation, communication, and control. Combining the two yields cell-silicon hybrids that pair biochemical breadth with engineered precision, enabling remote sensing and adaptive biomanufacturing. The development of such an integrated cell-silicon system has become possible through the establishment of bidirectional optical communication: light can modulate gene expression through optogenetic control, while fluorescent or luminescent protein reporters can relay gene expression readouts as electronic signals through photodiodes.^1–3^

Optogenetics uses light to control cellular processes, enabling rapid, reversible, and precise spatiotemporal regulation of gene expression—capabilities that chemical inducers often lack.^4^ Such control has been implemented at the transcriptional,^5–9^ translational,^10,11^ and post-translational levels.^12–14^ For example, the classic light-responsive CcaSR system, comprising a photoreceptor histidine kinase and a response regulator, enables reversible activation and repression of transcription.^6^ A remaining practical hurdle for cell-silicon control in typical laboratories is coupling real-time monitoring with dynamic illumination in standard microplate readers. Prior solutions achieve closed-loop regulation using chemostats with robotic sampling^15,16^ or single-cell feedback via automated microscopy in a mother machine.^17–19^ However, the complexity and cost of these platforms limit adoption and slow the design-build-test-learn cycle. To overcome these issues, Steel, H. et al.^20^ developed a custom miniaturized chemostat that enabled closed-loop optogenetic control, though the device’s utility is curbed by the sensor’s limited dynamic range.

The most common assay in synthetic biology grows cultures in well plates and uses a commercial microplate reader to measure optical density (OD_600_) and fluorescence. External LED-array stimulators have been built to modulate light,^21–25^ but typically require a human operator^26^ or a robotic stage to transfer samples to a microplate reader^27^ for measurement. Several all-in-one platforms combine illumination with onboard fluorescence and OD_600_ sensing for closed-loop control, but their optical readouts rely on fixed, custom hardware and lack the flexibility and precision offered by commercial microplate readers.^28,29^ For example, RT-OGENE uses a camera-based readout with fixed optical filters and illumination LEDs, which relies on image processing to estimate fluorescence and OD_600_, limiting the sensor’s dynamic range. The system also does not allow shaking, hindering growth and sustained protein expression.^28^ Similarly, the optoPlateReader relies on fixed LEDs, photodiodes, and optical filters and was optimized for blue-light stimulation and mAmetrine fluorescence readout.^29^ Using Lucifer Yellow dye as a benchmark fluorophore, the authors reported that optoPlateReader required roughly 80-fold higher signal levels for detection than a commercial plate reader, indicating substantially lower sensitivity. The authors also showed that replacing mAmetrine with green fluorescent protein (GFP) reduced the signal strength by about 70% because the emission filter strongly attenuated GFP emission. These results show that adapting such platforms to other fluorophores or optogenetic systems often requires major hardware changes and that performance depends strongly on filter selection, especially for reporters with small Stokes shifts.^2^ Due to the sensor limitations and the lack of an accurate gene expression model, the closed-loop controllers in both studies underperformed. As a result, progress toward practical cell-silicon hybrid systems has been limited, and there remains a need for an easy-to-use, reader-compatible platform for repeatable closed-loop studies in batch culture that accelerates the DBTL cycle.

In this work, we built the LED-Embedded Microplate for Optogenetic Studies (LEMOS) device as a prototyping platform and demonstrated its utility in the DBTL cycle for cell-silicon hybrid dynamical systems. LEMOS is a low-cost (≈$140), LED-embedded microplate that operates inside a commercial microplate reader and synchronizes illumination with measurement by blanking LEDs during reads. In batch *E. coli* cultures, we first mapped open-loop responses, then showed that proportional control tracks predefined set points, although with notable overshoot. Using our growth-dependent gene expression framework (GEAGS),^30^ we found that the observed dynamics arise from growth-dependent dead time in gene expression and predicted that adding integral and derivative actions would improve performance. Guided by the model, we designed and implemented Proportional-Integral (PI) and Proportional-Integral-Derivative (PID) controllers that improved both accuracy and response speed across conditions. By enabling feedback without leaving the microplate reader, LEMOS makes *in silico* feedback control practical in batch cultures, lowers the barrier to rapid DBTL, and is readily extendable to other bacterial strains and optogenetic mechanisms.

## Results

### LEMOS Device Design and Validation

The LEMOS device is designed to match the layout of a standard 96-well microplate, ensuring compatibility with conventional microplate readers (Figure 1A). The 3D-printed frame houses 16 slots for polydimethylsiloxane (PDMS) microwells, each with an individually addressable light-emitting diode (LED) mounted on the inner wall. The LEDs are controlled by an Arduino Nano33 IoT microcontroller with built-in Bluetooth, enabling wireless communication with an external computer. The microcontroller and the LEDs are powered by a Li-ion battery housed within the device, connected to a charging module and a three-position switch that toggles between “On”, “Off” or “Charging” modes. The detailed electronic components can be found in Supporting Information (SI) section “Running LEMOS Experiment” and the operation of the electronic components is described in Methods. To ensure sterility during cell culture experiments, PDMS sleeves are cast for each use via replica molding (see Methods). PDMS is a transparent, biocompatible elastomer well-suited for microwell fabrication due to its flexibility, chemical inertness, and ease of molding. Its optical transparency also enables fluorescence measurements during experiments. A 3D-printed mold (Figure 1C) was inserted into the LEMOS frame during casting (Figure 1B) to form wells shaped for incubation of 200 μL bacterial cultures (Figure 1D).

**Figure 1:**
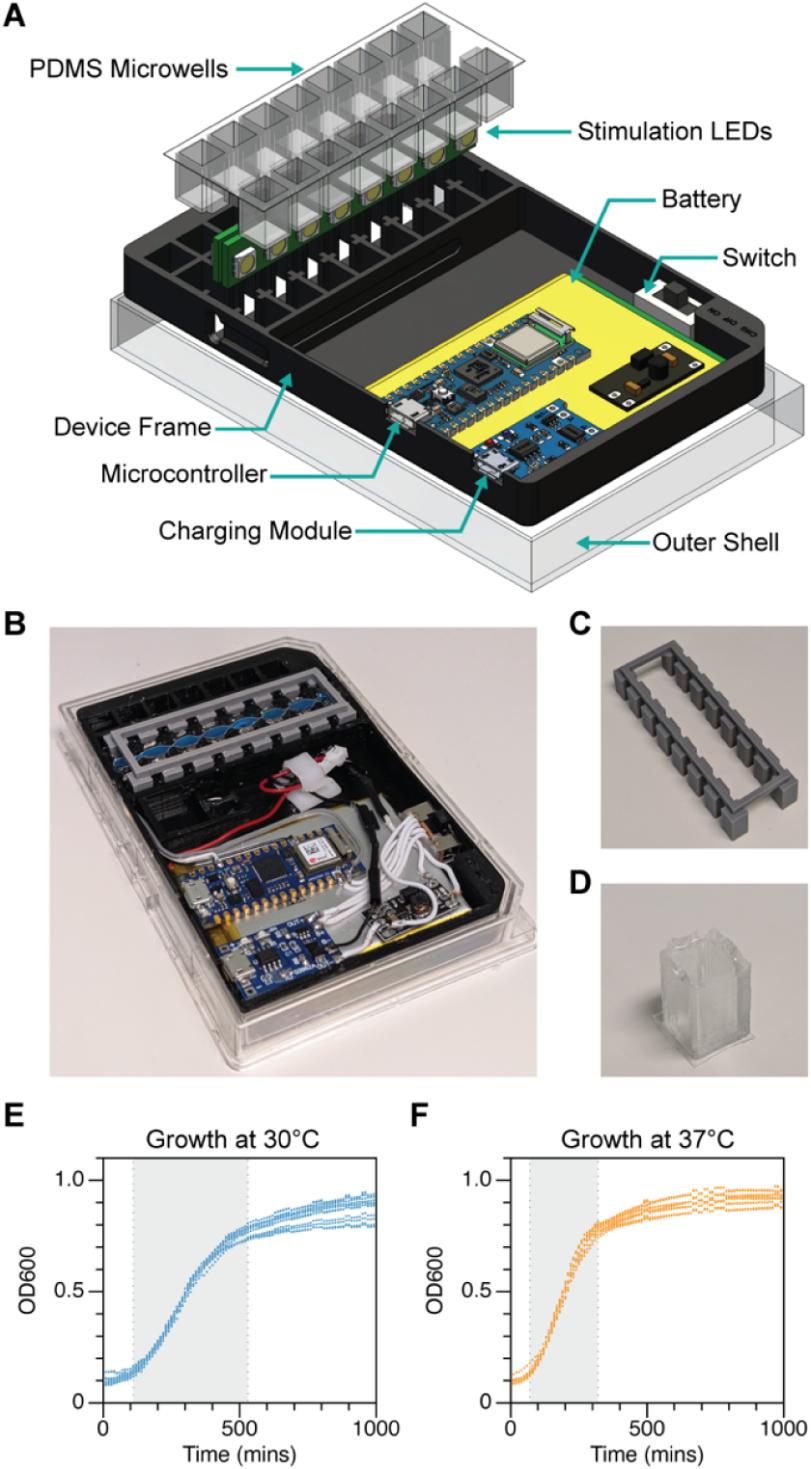
LEMOS device construction and cell growth characterization. (A) Exploded schematic of the LEMOS device showing PDMS microwells, stimulation LEDs, battery, switch, microcontroller, charging module, device frame, and outer shell. (B) Assembled device with the 3D-printed mold used to cast the PDMS microwells. (C) 3D-printed mold for microwell casting. (D) PDMS microwell cast in the LEMOS device. (E) Growth curves of *E. coli* in LEMOS at 30 °C and (F) 37 °C (*N* = 12 technical replicates). Gray shading marks the logarithmic (exponential) growth phase.

To determine whether the PDMS microwells support healthy bacterial growth and reliable measurement, we monitored bacterial cultures for 16 h in LEMOS. Continuous OD_600_ measurements were acquired using a standard microplate reader at 30 °C and 37 °C, under constant LED illumination. At both temperatures, cells exhibited logistic-like growth, with cultures at 30 °C showing a longer exponential phase than those at 37 °C. We therefore conducted all subsequent LEMOS experiments at 30 °C to maximize the dynamic measurement window before entry into the stationary phase. To assess whether LED-generated heat could perturb growth, we quantified temperature changes in the wells and compared growth at 30 °C under LED-on and LED-off conditions (SI section “Temperature effects of LED illumination in LEMOS wells”; Figure S7). These results showed that LED heating was minimal and did not measurably alter growth dynamics.

### System Characterization and Open-Loop Optogenetic Control

We used the CcaSR two-component system (TCS) optogenetic regulator system to control the expression of superfolder green fluorescent protein (sfGFP). The system consists of CcaS, a membrane-bound histidine kinase that senses green light, and CcaR, the corresponding response regulator^6^ (Figure 2A). Upon green light exposure, CcaS autophosphorylates and transfers the phosphate to CcaR, which dimerizes to activate the P_cpcG2_ promoter and induce sfGFP transcription. Red light reverses this process by promoting CcaS dephosphorylation, thereby repressing expression. To characterize the system, we performed a bulk fluorescence assay in a 96-well microplate. Cultures were incubated at 37 °C in a custom light box under green light, red light, and dark conditions (see Methods). Fluorescence was measured manually every hour for 6 h, using a microplate reader. The system functioned as expected in *E. coli* MG1655, with sfGFP activation under green light, repression under red light, and intermediate expression in the dark due to basal promoter activity (Figure 2B).

**Figure 2:**
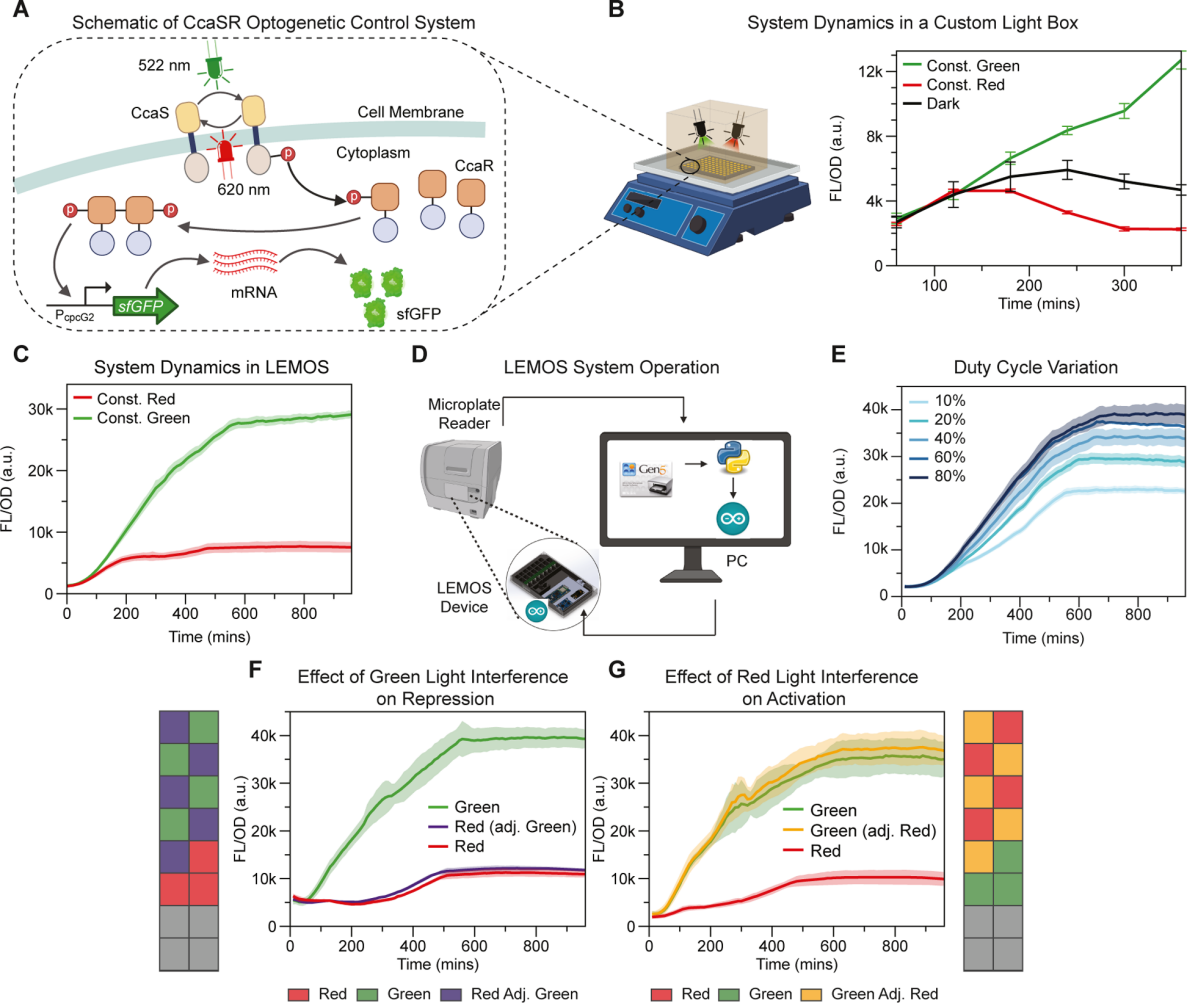
Optogenetic system characterization in LEMOS and open-loop control dynamics.(A) Schematic description of the CcaSR two-component optogenetic system: green light (522 nm) activates CcaS/CcaR to upregulate P_cpcG2_-sfGFP expression, and red light (620 nm) downregulates the expression. (B) Activation-repression dynamics in sfGFP fluorescence/OD_600_ in a standard 96-well plate (*n* = 4) under constant LED illumination using a custom light box; the plate is manually transferred to the microplate reader for measurement every hour for 6 hours. The error bars indicate standard deviation. (C) Same assay in the LEMOS device (*N* = 3) for 16 h, with one measurement every 10 min. Solid lines indicate means; shaded bands indicate standard deviations. (D) LEMOS operation schematic. During the 16-h time course, the device remains in the microplate reader. The reader measures sfGFP fluorescence and OD_600_ and streams data to a computer, which handles timekeeping and operates Arduino routines that command the onboard microcontroller to temporarily disable LED illumination during each measurement. (E) Open-loop responses in LEMOS to varying green-light duty cycles (10–80%, *N* = 3). Solid lines indicate means; shaded bands indicate standard deviations. (F) Effect of green-light interference on repression. Wells were continuously illuminated with green or red light. In the plate layout, green boxes indicate green-light wells (N = 4), purple boxes indicate red-light wells (N = 5) adjacent to green-light wells, and red boxes indicate red-light wells (N = 3) not adjacent to any green-light wells.(G) Effect of red-light interference on activation. In the plate layout, red boxes indicate red-light wells (N = 4), yellow boxes indicate green-light wells (N = 5) adjacent to red-light wells, and green boxes indicate green-light wells (N = 3) not adjacent to any red-light wells. N denotes the number of technical replicates; n denotes the number of biological replicates.

To enable continuous measurement, we inoculated the same cell lines within the microwells of the LEMOS device and incubated them in a microplate reader. One key requirement for this setup was to prevent interference from the LEMOS LEDs with the optical measurements taken by the microplate reader. To address this, we synchronized LEMOS with an external computer, which handled timekeeping and communicated with the device via Bluetooth. The microplate reader was programmed to record OD_600_ and fluorescence every 10 min. During each interval, the LEDs were programmed to turn off for the first and last minute to avoid light interference during measurements. As shown in Figure 2C and 2D, this setup allowed real-time monitoring of sfGFP expression dynamics under both green and red light over a 16-h time course, with measurements taken every 10 min.

To evaluate the system’s response to actuating parameters, we characterized its dynamics under varying duty cycles (see Methods). Duty cycle refers to the fraction of time within an interval during which green light is applied, with red light used for the remainder. Our control period was set to 10 min; then the LEDs were on for 8 min every interval, during which we varied the proportion of green and red light. A 100% duty cycle corresponds to constant green light, while 0% corresponds to constant red light. As shown in Figure 2E, we designed an experiment to test duty cycles ranging from 10% to 80%. The relative sfGFP expression levels exhibited a clear positive correlation with the duty cycle, indicating sensitive system output responses (sfGFP signal) to the actuating parameter (duty cycle).

Finally, to assess interwell crosstalk, we performed open-loop experiments in which adjacent wells were continuously exposed to either red or green light. Red-light wells adjacent to green-light wells [Red (adj. Green)] behaved similarly to red-light wells without green-light neighbors, with only minor differences and no qualitative change in repression dynamics (Figure 2F). Likewise, green-light wells adjacent to red-light wells [Green (adj. Red)] closely overlapped with green-light wells without red-light neighbors, indicating a minimal effect on activation dynamics (Figure 2G). Together, these results show that interwell crosstalk is minimal and does not measurably affect light-mediated activation or repression.

### Closed-Loop Proportional Feedback Control for Set Point Tracking

Next, we implemented closed-loop cell-silicon feedback control for set point tracking using the LEMOS device. Set point tracking is a standard control task that drives a measured system variable to a defined value. In this setup, as shown in Figure 3A, gene expression in living bacterial cells serves as the process plant, with sfGFP fluorescence as the output. The microplate reader senses this output and transmits the data to an external computer acting as the controller. Based on the measured signal, the computer calculates control actions and sends commands to the LEMOS microcontroller, which adjusts the LED duty cycle (the actuator) to regulate gene expression via optogenetic input. In our case, the set point is a predefined FL/OD_600_ intensity that signifies the amount of sfGFP expressed per cell on average.

**Figure 3:**
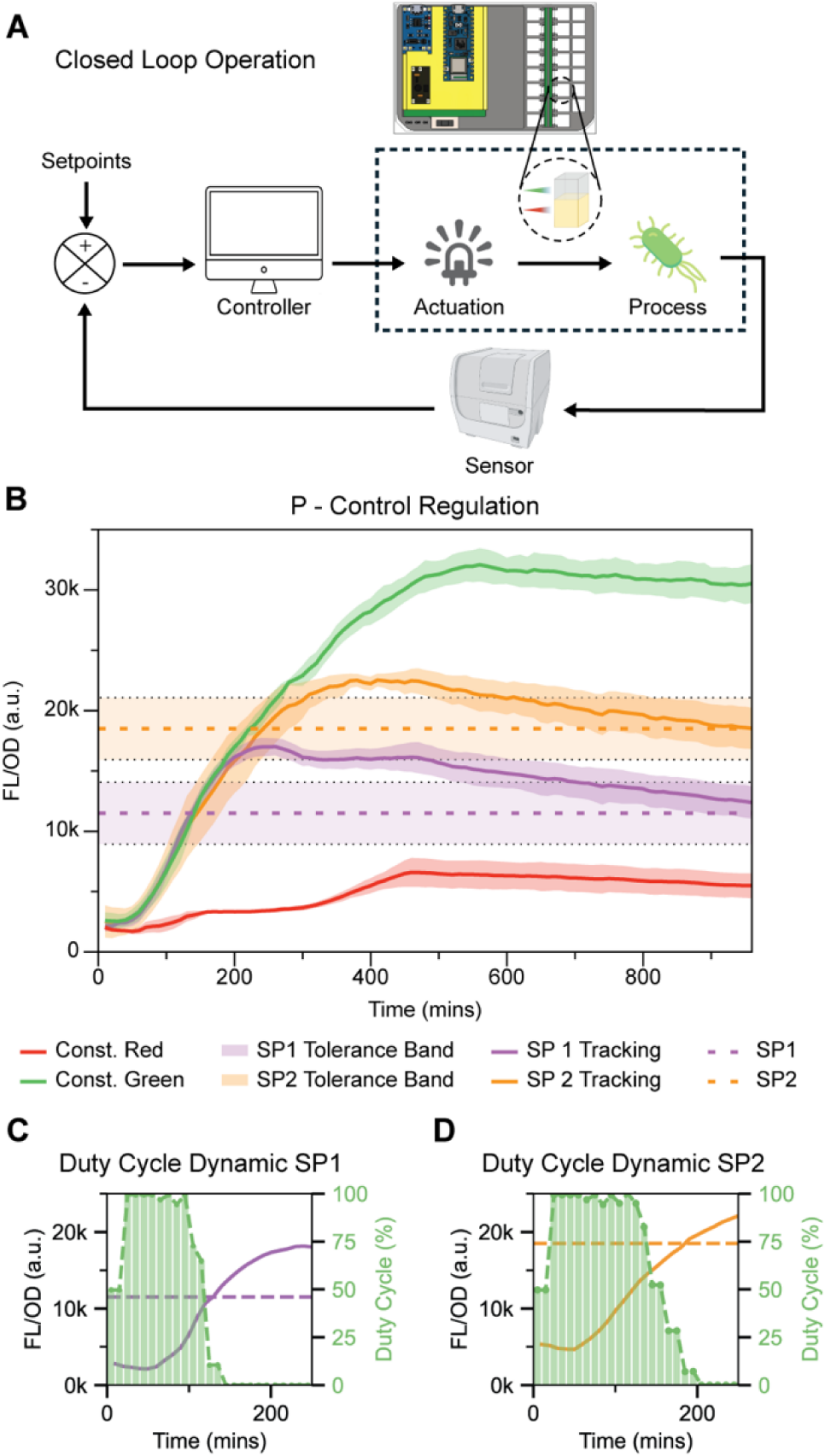
Closed-loop control in LEMOS (A) Schematic of closed-loop operation: the microplate reader functions as the sensor that measures FL/OD, the computer functions as the controller, and the LEMOS device actuates LEDs to regulate gene expression. The process plant is gene expression in living bacterial cells. (B) Proportional (P) control tracking of FL/OD set points. Constant-light references are green and red (*N* = 4, *n* = 2). The 2 set point tracking runs are described by yellow and purple lines (*N* = 9, *n* = 2, total 18 trajectories). Solid lines indicate the mean FL/OD; shaded bands indicate standard deviations. Dotted lines mark set points (SP1 = 1.15 × 10^5^ a.u., SP2 = 1.85 × 10^5^ a.u.); shading around set points indicates ±10% tolerance. (C–D) Duty-cycle command for representative SP1 and SP2 runs; each panel shows a single well trajectory. N denotes the number of technical replicates; n denotes the number of biological replicates.

To evaluate set point tracking, we assigned two distinct set points to two groups of microwells, corresponding to the orange and purple dotted lines in Figure 3B. The controller continuously compared the measured fluorescence to each well’s assigned set point and computed the LED duty cycle for the next 10-min control interval. We first implemented proportional control (P-control), where the duration of green light exposure in each cycle (*t_green_*) is computed according to eq 1:

1 $$t_{green}=K\cdot \left(e\left(t\right)\right)$$

Here, *e*(*t*) is the error between the measured FL/OD_600_ signal and the set point at time *t*, and *K* is the proportional gain that determines the controller’s response sensitivity. The remaining portion of the 8-min control interval not occupied by green light was filled with red light. Because no measurement is available at *t* = 0, the controller initializes with a default 50% duty cycle for the first two intervals. We chose the value of *K* = 0.064 min/a.u., based on an order-of-magnitude estimate. Figures 3C and 3D illustrate how the duty cycles change over time for the two set points. In these plots, the height of each green bar represents the fraction of green light (i.e., the duty cycle) applied during each 8-min control period. As expected, the duty cycle decreases rapidly as the measured sfGFP approaches the set point, eventually reaching 0% once the signal reaches the set point. Notably, if the signal drops below the set point further in time, the P-controller action kicks in to increase the duty cycle (Figure S10). As shown in Figure 3B, both set points, FL/OD = 11.5e4 (SP1) and 18.5e4 units (SP2), were successfully tracked by the P-controller. However, despite the fast response time of the electronic actuators, both trajectories exhibited notable overshoot before gradually converging to their respective set points.

### Growth-Dependent Dead Time Leads to Signal Overshoot

To improve controller performance, we used a model-guided strategy to address a key question: Does the consistent overshoot under P control arise from standard gene-expression kinetics or from an unexpected mechanism? To test this, we constructed a deterministic kinetic model of the system’s dynamics.

As shown in Figure S11, because growth is non-constant in batch culture, a traditional growth-independent effective model fails to recapitulate the observed dynamics. To capture batch-culture behavior, we adopted our previously published Gene Expression Across Growth Stages (GEAGS) model framework:^30^ we fit a logistic function (Figure 4A) to the growth data from the closed-loop experiment in Figure 3 and modeled gene expression rates as functions of the instantaneous growth rate. Model details are provided in SI section “GEAGS model”. With these modifications, the optogenetic GEAGS simulations closely matched the experiments, accurately reproducing sfGFP dynamics under both constant green and red-light inputs (Figure 4B).

**Figure 4:**
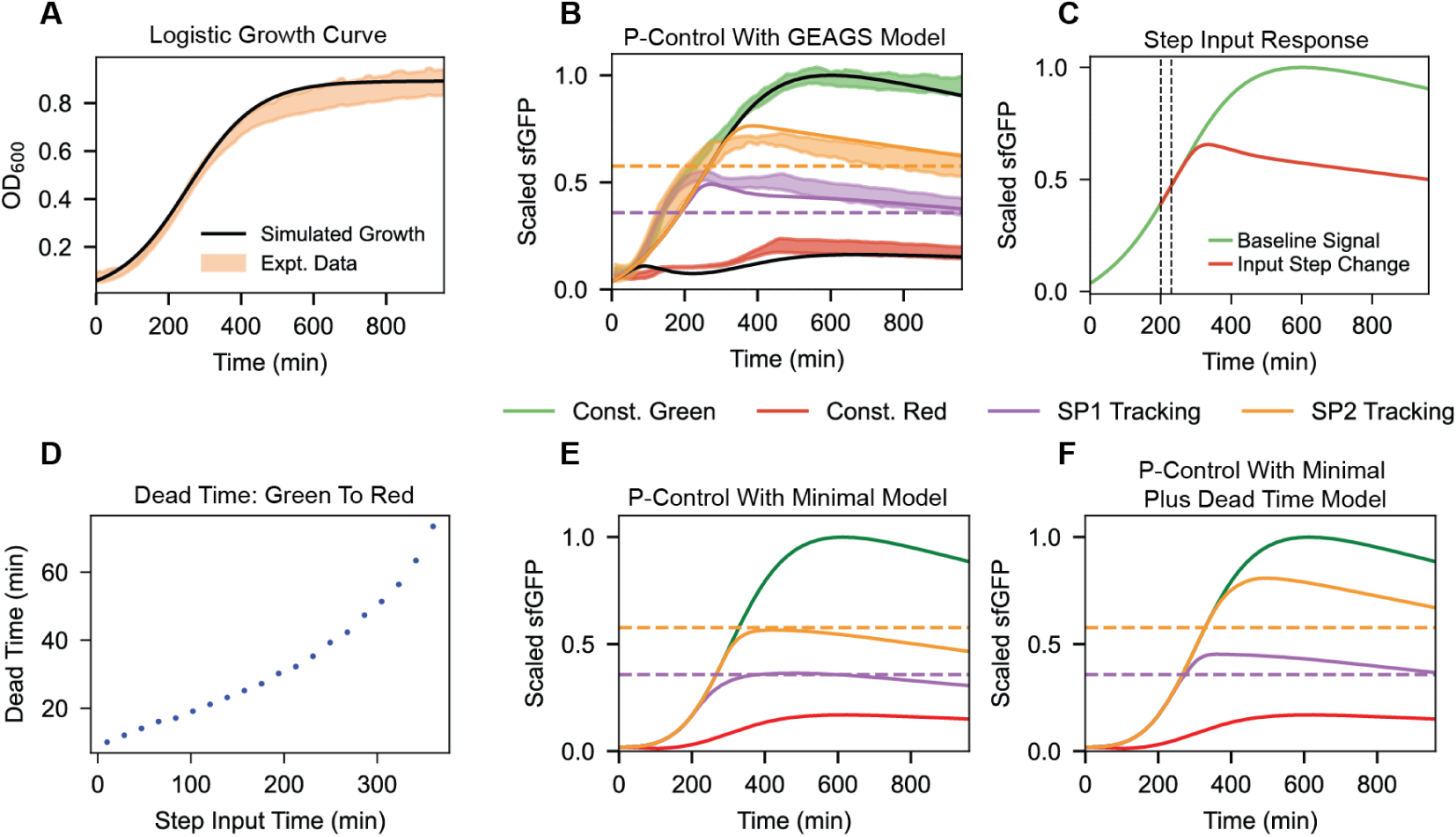
GEAGS growth-aware modeling explains dead time and P-control behavior. (A) Simulated logistic growth overlaid with measured OD_600_ (*N* = 18, *n* = 2). (B) P-control simulations with the GEAGS model overlaid on experimental set point-tracking data. Solid lines indicate model simulation, and shaded regions indicate experimental data (*N* = 9, *n* = 2 for set point tracking). Dashed colored lines represent the predetermined set point. (C) Step-response simulation with the GEAGS model illustrating dead-time estimation after a green-to-red actuator-light switch at t = 200 min. Vertical dashed lines mark the input step and the onset of response; their separation defines the dead time. (D) Response dead time of the green-to-red switch increases over time in batch culture. (E) P-control dynamics simulated with a minimal model omitting gene expression dynamics associated with time delay (SI section “Minimal GEAGS Model”), showing minimal system overshoot. (F) P-control dynamics simulated with the minimal-plus-dead-time model recapitulates the observed overshoot. N denotes the number of technical replicates; n denotes the number of biological replicates.

To investigate the consistent overshoot observed in the P-control regulation experiment, we simulated the system by using the GEAGS model framework. The simulation results closely matched the experimental data (Figure 4B), capturing the overshoot behavior. This agreement suggests that the overshoot arises from well-understood mechanisms intrinsic to the system. We hypothesize that it is primarily driven by inherent delays in the gene expression process, such as translation, protein folding, and maturation.

To quantify system delays, we measured the dead time in the optogenetically controlled circuit. Dead time is the interval after an input change during which the output shows no detectable response; here, it refers to the lag between an LED color shift and the resulting measurable change in the FL/OD. We first simulated a baseline FL/OD trajectory under constant green light (Figure 4C, green curve). We then introduced step changes from green to red at multiple time points and recorded how long it took for the FL/OD signal to deviate from the baseline. At each time *t*, as shown in Figure 4D, the dead time increases over time, indicating a gradual decline in system responsiveness as the batch culture approaches the stationary phase. This trend is consistent with the general understanding that gene expression slows as cellular resources are depleted during growth arrest, in addition to substantially reduced protein and mRNA turnover via dilution, resulting in increasingly sluggish system dynamics.

To isolate the effect of dead time on closed-loop control dynamics, we first simulated a delay-free minimal model that assumes minimal gene-expression delay and omits protein folding and two-component switching (SI Section “Minimal GEAGS Model”). As expected, the closed-loop response showed negligible overshoot (Figure 4E). To examine the role of dead time in isolation, we then introduced a minimal-plus-dead-time model by inserting the experimentally estimated dead time *t_dt_* (*t*) at each time step (Figure 4D). Under P control, the controller at time *t*_*i*_ computes the duty cycle based on the sfGFP value at a prior time *t_i_* – *t_dt_* (*t_i_*), emulating the delay and effectively shifting the perceived system state. This model reproduces the experimental overshoot (Figure 4F), indicating that the overshoot arises from biological sluggishness: while sensing, computation, and actuation are millisecond-scale electronically, transcription, translation, and fluorescent-protein maturation limit the overall response.

### Implementation of PI and PID Feedback Control

Since proportional control consistently produced overshoot driven by dead time and slow gene-expression dynamics, we next evaluated proportional–integral (PI) and proportional–integral–derivative (PID) controllers. In control theory, PI control eliminates steady-state error by integrating past errors, while PID adds derivative action to improve transient response by anticipating changes in the error.^31,32^ These strategies are particularly useful for slow or delay-prone systems, where proportional control alone is insufficient. We implemented both PI and PID schemes to test whether they could achieve more accurate set-point tracking and faster convergence.

We first examined a PI controller to compensate for the accumulated error. The integral term sums past errors, which can reduce overshoot and improve tracking accuracy.^32^ In our implementation, the controller updated the duty cycle once every 10 min according to eq 2:

2 $$t_{green}=K\cdot \left(e\left(t\right)+\frac{1}{\tau_{I}}\cdot \int e\left(t\right)dt\right)$$

Here, *τ_I_* is the integral time constant, which scales the accumulated error *e*(*t*). Using the GEAGS model with the same parameters as in Figure 4A and 4B simulations, we optimized PI controller gains and selected *τ_I_* = 1500 min and *K* = 0.013 min/nM. Simulations showed that the PI controller markedly reduced overshoot and achieved convergence at both set points (Figure 5A), in contrast to P control (Figure 4B). We then implemented this model-guided design on the LEMOS plate, which confirmed consistently low overshoot for both set points (Figure 5B). Tracking at SP2 was slightly less optimal, with minor overshoot. As indicated in Figure 5E, the duty-cycle update appears insufficiently responsive, contributing to this residual overshoot and suggesting room for further controller refinement.

**Figure 5:**
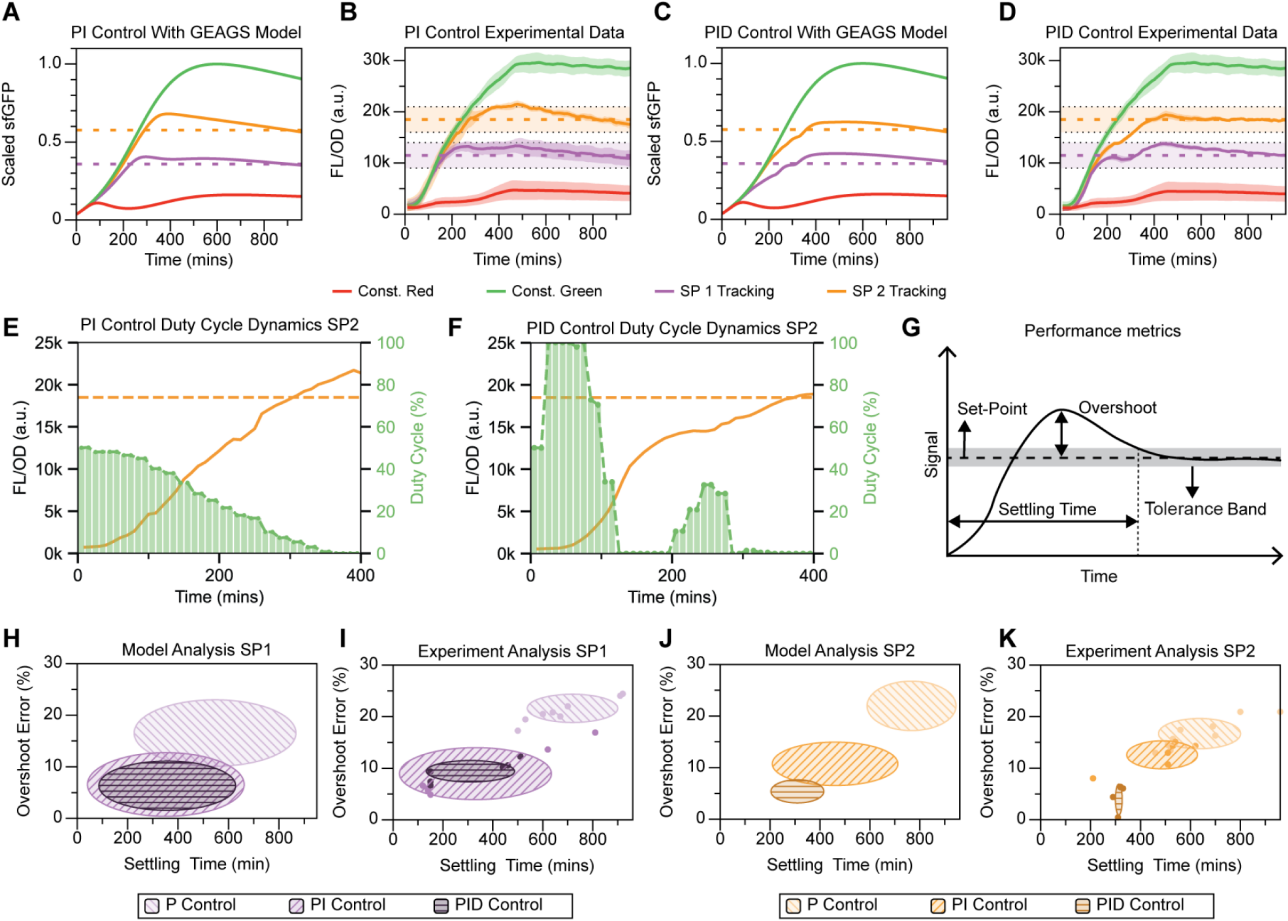
Model-guided PI and PID regulation. (A) GEAGS-based PI control simulations with tuned gains. (B) PI control experiments in LEMOS: constant-light references (*N* = 14, *n* = 6 each) and set point tracking at SP1 and SP2 (*N* = 6, *n* = 2 each). (C) GEAGS-based PID control simulations with tuned gains. (D) PID control experiments in LEMOS: constant-light references (*N* = 14, *n* = 6 each) and set point tracking at SP1 and SP2 (*N* = 6, *n* = 2 each). (E–F) Duty-cycle command for representative SP2 tracking with PI and PID controllers; each panel shows a single well trajectory. (G) Definition of performance metrics: overshoot (%) and settling time to enter and remain within a ±10% band around the set point. (H–K) Comparison of P, PI, and PID performance from model predictions (H, J) and experiments (I, K) for SP1 and SP2. The ovals indicate variability in each control strategy: the horizontal radius reflects ±1 standard deviation of Settling Time, and the vertical radius reflects ±1 standard deviation of Overshoot Error (%), calculated across replicates. N denotes the number of technical replicates; n denotes the number of biological replicates.

To further improve set-point tracking performance, we implemented a proportional-integral-derivative (PID) control strategy. In addition to the proportional and integral terms, PID control includes a derivative term that responds to the rate of change of the error according to eq 3:

3 $$t_{green}=K\cdot \left(e\left(t\right)+\frac{1}{\tau_{I}}\cdot \int e\left(t\right)dt+\tau_{D}\cdot \frac{de\left(t\right)}{dt}\right)$$

Here *τ_D_* is the derivative time constant, which sets how much the slope of the error contributes to the control signal. The derivative term anticipates future error from its current rate of change.^32^ Using the GEAGS model with the same parameters as in Figure 4A and 4B simulations, we optimized the PID controller parameters and selected τ*_I_* = 1500 min, *K* =0.032 min/nM, and *τ_D_* = 120 min. Simulations showed that PID control (Figure 5C) substantially reduced overshoot and improved convergence for SP2 relative to PI control, with only minor improvement for SP1. We implemented this design on the LEMOS device, and the experiments matched the predictions: PID enhanced tracking for SP2 with limited added benefit for SP1 (Figure 5D). As shown in Figure 5F, the duty-cycle trajectory exhibits a stair-step profile. An early rapid rise in signal (before ∼100 min) prompted a strong reduction in green light. As the signal’s rate of increase slowed, the controller raised the duty cycle between 200 and 270 min, followed by another sharp reduction near 300 min. This pattern is consistent with derivative action operating on a slow, sampled system, yielding anticipatory but discretely timed adjustments.^31^

To compare controller performance, we defined two performance metrics: overshoot and settling time (Figure 5G). With activation at *t* = 0 and a 10% tolerance band around each set point, overshoot error is the maximum deviation of the sfGFP signal from the set point, and settling time is the time required for the signal to reenter the tolerance band after the overshoot. We visualized performance on a map with overshoot on the *y*-axis and settling time on the *x*-axis, where strategies closest to the origin achieve faster responses and more accurate tracking.

For SP1, we evaluated P, PI, and PID control. In GEAGS simulations, we generated 50 trajectories by perturbing parameters ±15% around their fitted values and recorded the resulting metrics. The transition from P to PI control clearly improved both speed and accuracy, whereas PID yielded little additional benefit (Figure 5H). The experimental analysis showed the same pattern (Figure 5I). Applying the same procedure to SP2 in both simulations (Figure 5J) and experiments (Figure 5K) showed a consistent progression in performance from P to PI to PID. We also tested model robustness to perturbations in controller gains for all three controllers at both set points and observed the same progression from P to PI to PID (SI Figure S13), indicating that the model is robust to variations in controller gains.

Overall, these results indicate that the derivative action was dispensable for SP1 tracking but became essential for SP2. We attribute this difference to growth-phase effects: SP1 was reached earlier, during logarithmic growth, when the system dead time was small and responsiveness was high, whereas SP2 was reached later, when the system was more sluggish, making derivative control critical for maintaining responsiveness.

## Discussion

Our work effectively showcases that the LEMOS device makes closed-loop optogenetic control as routine as reading OD and fluorescence in a commercial microplate reader. By embedding LEDs in a microplate and blanking illumination during measurement windows, this platform preserves reader sensitivity and removes transfer between stimulation and readout. In batch *E. coli* cultures, proportional control tracked early set points but overshot as growth slowed. A growth-aware GEAGS model explained this behavior as growth-dependent dead time and guided PI and PID designs that improved accuracy and settling across conditions.

Mechanistically, gene expression in batch culture behaves as a process plant whose effective delay increases as cultures approach the stationary phase. Transcriptional and translational latencies, protein maturation, and biomass dilution slow the apparent response relative to the controller’s sampling period and set point changes. Since responsiveness depends on the growth phase, a controller tuned for one set point or phase may be suboptimal in another. This highlights the multiscale nature of closed-loop gene regulation, where population dynamics shape molecular kinetics and determine which control structures and gains provide robust performance.

The LEMOS device makes closed-loop optogenetic control practical inside the same microplate reader used for routine OD and fluorescence, lowering the barrier to model-informed DBTL and revealing growth-phase effects that are often hidden in chemostats or single-cell rigs. Open-loop kinetics collected with LEMOS guided the development of a growth-dependent deterministic model that accurately captures the hybrid system’s dynamics and guides controller design. To the best of our knowledge, this is the first end-to-end DBTL cycle to achieve functional closed-loop optogenetic control in batch culture. Together, the accessible hardware and growth-aware model make DBTL for closed-loop cell-silicon systems a routine workflow, enabling rapid iteration and more reliable design. Both the device and the modeling framework are generalizable and can be extended to other hosts and optogenetic actuators.

The current LEMOS device offers three illumination channels (red, green, and blue; RGB). In this work, we used the green and red channels to drive the CcaSR system. The device is ready to evaluate systems using blue-light actuators, such as EL222,^33,34^ iLID,^35^ or VVD.^36^ Variants can also be built to incorporate different LED lights to test optogenetic systems that are stimulated by, for example, near-infrared light^37^ and violet light.^38^ Future versions of LEMOS will explore replacing the current PLA base with more heat-resistant, autoclavable PLA formulations that can withstand steam sterilization.^39^ This could simplify device reuse by eliminating the need to cast new PDMS microwells for each experiment. To increase the throughput, we will also work on adding more wells to the device. On the control algorithm side, because response speed varies with growth, controller gains should adapt accordingly. We will explore gain scheduling based on OD or online growth-rate estimates,^40^ add antiwindup and explicit disturbance-rejection tests, and, leveraging the calibrated GEAGS model,^30^ implement model predictive control (MPC) that uses short-horizon forecasts to update inputs across phases and set points. In addition, we will also use LEMOS to study layered control in synthetic biology^41^ by combining molecular feedback or feedforward circuits with cell-silicon control and quantify how layering affects set-point tracking and disturbance rejection performance. Together, these extensions will expand LEMOS beyond batch culture and improve its robustness across hosts and circuits.

## Materials and Methods

### LEMOS Device and PDMS Microwell Fabrication

The LEMOS device integrates the electronic components necessary for optical stimulation and wireless communication with an external computer running a Python script. It is built on a 3D-printed PLA frame designed to fit within a standard single-well plate and currently supports 16 microwells with independently controlled LEDs. A full list of components is provided in the SI section “Bill of Materials”. The 16 LEDs in the LEMOS device are connected via a single I^2^ C bus controlled by an Arduino Nano33 IoT microcontroller, which also includes a Bluetooth module for wireless communication with an external computer. Both the microcontroller and LEDs are powered by an onboard Li-ion battery. Each LED is individually addressable over the I^2^ C bus, with user-defined control over color and intensity. The emitted wavelengths are determined by the number and type of discrete diodes in each multicolor LED. In this design, we selected commercial LEDs containing three diodes with emission peaks at 620–625 nm, 522–525 nm, and 465–467 nm. The green and red channels lie within the broad activation (≈535 nm) and deactivation (≈620–670 nm) bands reported for the CcaSR system,^42^ so these commercially available wavelengths are compatible with the regulating spectra of CcaSR despite their small offsets from the canonical peak values. These LEDs use discrete pulse-width modulation (PWM) for intensity control, which cannot be continuously varied during the experiments. Accordingly, we operated the LEDs at a fixed intensity and used duty cycle as the actuating variable; the fixed intensity was selected to provide sufficient optical stimulation while minimizing heat generation and interwell light crosstalk. PDMS microwells were fabricated in the LEMOS device using replica molding by casting PDMS around a 3D-printed mold insert. A 1:10 ratio of the curing and base agents of RTV 615 (Momentive Inc.) was mixed thoroughly and degassed under vacuum in a desiccator for up to 1 h to remove air bubbles. It is critical to remove the air bubbles from the PDMS before casting since the bubbles change the optical transmission properties of the bottoms of each well, leading to nonuniform baseline OD measurement across wells. Note that in the experiments, the variation in the OD measurement was quantified by measuring OD in each well with blank media before the experiment. The degassed PDMS mixture was then poured into the LEMOS device, into which a 3D-printed PLA mold was inserted to define the shape of the wells. The PDMS in the device was cured overnight at room temperature, after which the PLA mold was carefully removed. After each experiment, the culture in the PDMS microwells was discarded, and new PDMS microwells were cast, to ensure sterility and reuse of the LEMOS device.

### Experimental Software Programming

LEMOS operation requires data readout from the microplate reader after each control period of 10 min. On a computer running Biotek Gen5, as in this study, a custom Python script monitors the export directory after each cycle and collects the exported data, storing fluorescence (FL) and optical density (OD_600_) values in separate CSV files. If the CSV files do not exist, the script creates them automatically. To avoid export errors from the microplate reader, the script deletes each exported file after processing. Interaction with the Gen5 user interface is automated by using the pyautogui library in Python, which is used to select the appropriate interface elements required for initiating each cycle. Exported CSV files are parsed using a custom datafile_manager module that extracts the relevant measurements and formats them for the closed-loop control calculations (see GitHub for implementation details). All of the experiments carried out in LEMOS follow this protocol. If an alternative microplate reader is used, and the plate reader software is different from Biotek Gen5, the overall workflow remains unchanged; however, the user must configure the corresponding software to periodically measure and export the measurement data as CSV files to a specified directory. On the Python side, the datafile_manager module must be updated to parse the specific CSV format produced by the software, and the reference images used by pyautogui must be updated to match the graphical user interface of the reader software. As long as a periodic automated export is supported by the plate reader software, identical data handling and closed-loop control calculations can be performed.

### Custom Light Box Experiments

The experiment shown in Figure 2B used a custom light box built by attaching red (Broadcom Limited #HLMP-3762, 626 nm) and green (Kingbright #WP7083ZGD/G, 525 nm) LEDs to the top of a cardboard box. This box was placed over a 96-microwell plate containing cultures, which were maintained on a shaking plate at 37 °C for proper growth. For each condition, either the red or the green LED was continuously on throughout the experiment. Fluorescence (excitation: 485 nm; emission: 528 nm) and optical density (OD_600_) were measured every 60 min using a BioTek Synergy H1 microplate reader.

### Strains and Culture

The experimental strain was constructed by transforming *E. coli* MG1655 with plasmid pNO286–3 (Addgene #107746) and plasmid pSR58.6 (Addgene #63176). As a negative control for autofluorescence, we constructed an empty backbone by deleting the functional gene cassettes, including P_cpcG2_ and sfGFP, using standard inverse PCR. Cells were plated on LB agar containing 50 μg/mL spectinomycin and 25 μg/mL chloramphenicol and incubated overnight at 37 °C, after which the plates were stored at 4 °C in the dark to minimize light exposure. All experiments were performed in Minimal M9CA Broth (Teknova #M8010) supplemented with 50 μg/mL spectinomycin and 25 μg/mL chloramphenicol. For each condition, a single colony was inoculated into 2 mL of media in a sterile culture tube and grown overnight at 37 °C with shaking at 220 rpm. To prevent unintended light exposure, all cultures were maintained in foil-wrapped tubes. A 100 μL aliquot of the overnight culture was transferred into 2 mL of fresh media in a sterile culture tube and incubated for 4 h at 37 °C with shaking at 220 rpm. By the end of this period, cultures reached an optical density (OD_600_) of 0.4–0.6. Experimental cultures were then prepared by diluting the preculture to an OD_600_ of 0.1, and 200 μL of each condition was added to the PDMS sleeves of the LEMOS device. The device was sealed with a Breath-Easy film (USA Scientific #9123–6100) and loaded into the microplate reader to initiate the experiment. Fluorescence (excitation: 485 nm; emission: 528 nm; gain: 50) and optical density (OD_600_) were measured every 10 min using a BioTek Synergy H1 microplate reader, incubated at 30 °C with maximum linear shaking.

### Statistical Analysis

All single colonies were picked randomly from agar plates, where the cells were transformed with purified circular DNA using antibiotics as selection markers. No manual group allocation methods were used. Each plate resulted from an independent transformation, and all colonies were assumed to be biological replicates. No data were excluded from the analyses in experiments presented in Figure 1E, 1F, 2B, 2C, 2E, 2F, 2G, 3B, 3C, 3D, 4A, 4B, 5B, 5D, 5E, 5F, 5I, 5K, S11, and S12.

## Supplementary Material



## Data Availability

All data needed to evaluate the conclusions in the paper are present in the paper and/or the Supporting Information. The source code for all the simulations is available at the following GitHub repository: https://github.com/synbiosystems/LEMOS-models-and-data. Information about the LEMOS device can be found publicly at the following GitHub repository: https://github.com/kpochana/LEMOS.

## Notes

The Supporting Information contains: Bill of Materials (Table S1); LEMOS-plate reader interface workflow (Figures S1–S6); additional experimental data on device assessment (Figures S7–S10); and description of the dynamic models and parameters (Figures S11–S13, Tables S2–S7) (PDF)
