# Supplementary material for: Closed-Loop Optogenetic Control in a Microplate Reader
Source: ACS Synth Biol. 2026 May 25;15(6):2356–65. doi: 10.1021/acssynbio.6c00003 (PMC13288875; doi:10.1021/acssynbio.6c00003)
Supplement: Supplementary file 1 [file sb6c00003_si_001.pdf]

Supporting Information for:

Closed-loop Optogenetic Control in a Microplate Reader

Hari R. Namboothiri[1†], Krishna Pochana[2†], Bhavya Jaiswal[1], Azita Emami[2], Chelsea Y. Hu[1,2 *]

1. Department of Chemical Engineering, Texas A&M University, College Station, TX 77843
2. Division of Engineering and Applied Science, California Institute of Technology, Pasadena, California, 91125

†. These authors contributed equally.
* Correspondence: Chelsea Y. Hu, Department of Chemical Engineering, Texas A&M University, College Station, TX 77843, Email: chelsea.hu@tamu.edu

Bill of Materials

Table S1. Bill of materials for LEMOS device construction.

Sr.no	Item	Link	Notes
1	Arduino Nano33 IoT	https://www.amazon.com/Arduino-Nano-33-IoT/dp/B07VW9TSKD	One runs the device, other is used with the central computer
2	Lipo battery	https://www.amazon.com/gp/product/B0BXNDNTRP	Battery for device
3	Battery management board	https://www.amazon.com/gp/product/B071RG4YWM	Handles battery charging
4	Voltage converter board	https://www.amazon.com/gp/product/B09D3G96KZ	Steps battery voltage to logic-level voltage
5	WS2812B LED strip	https://www.amazon.com/gp/product/B07BTTY4FL	LEDs for optical stimulation
6	DP3T switch	https://www.amazon.com/uxcell-Horizontal-Switch-Terminals-Latching/dp/B07H3RPD23	Power and mode switch for device
7	22 AWG solid core wire	https://www.amazon.com/TUOFENG-Electronic-Prototyping-Circuits-Breadboarding/dp/B07TX6BX47	Hookup wire
8	SUNLU 1.75mm PETG 1kg - Black	https://www.amazon.com/SUNLU-Official-Elite-Filament-1-75mm/dp/B0CFLW4LCJ	Black PETG filament for device frame

Running LEMOS experiment

Gen5 operation:

a) LEMOS experiment initialization. Open the ‘closed loop protocol’ Gen5 file.

b) Select ‘Create experiment and read now’ (Fig. S1) and save the experiment file in the folder where the FL and OD values will be exported (normally the file named Datafile within the experiment directory).

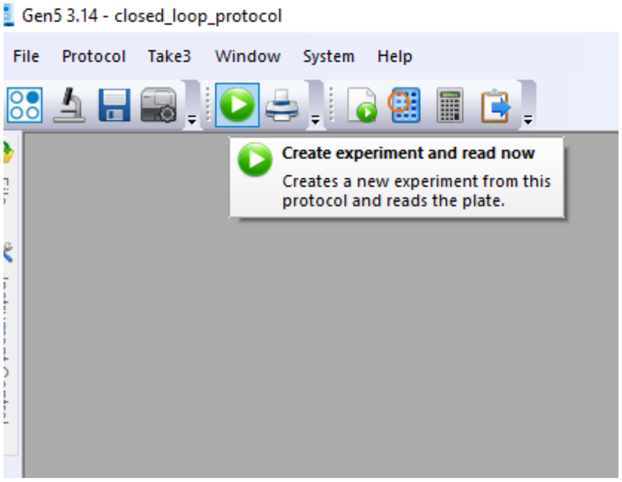

Fig. S1 Create experiment for LEMOS

c) Specify the location of the file exported from Gen5 (Fig. S2).

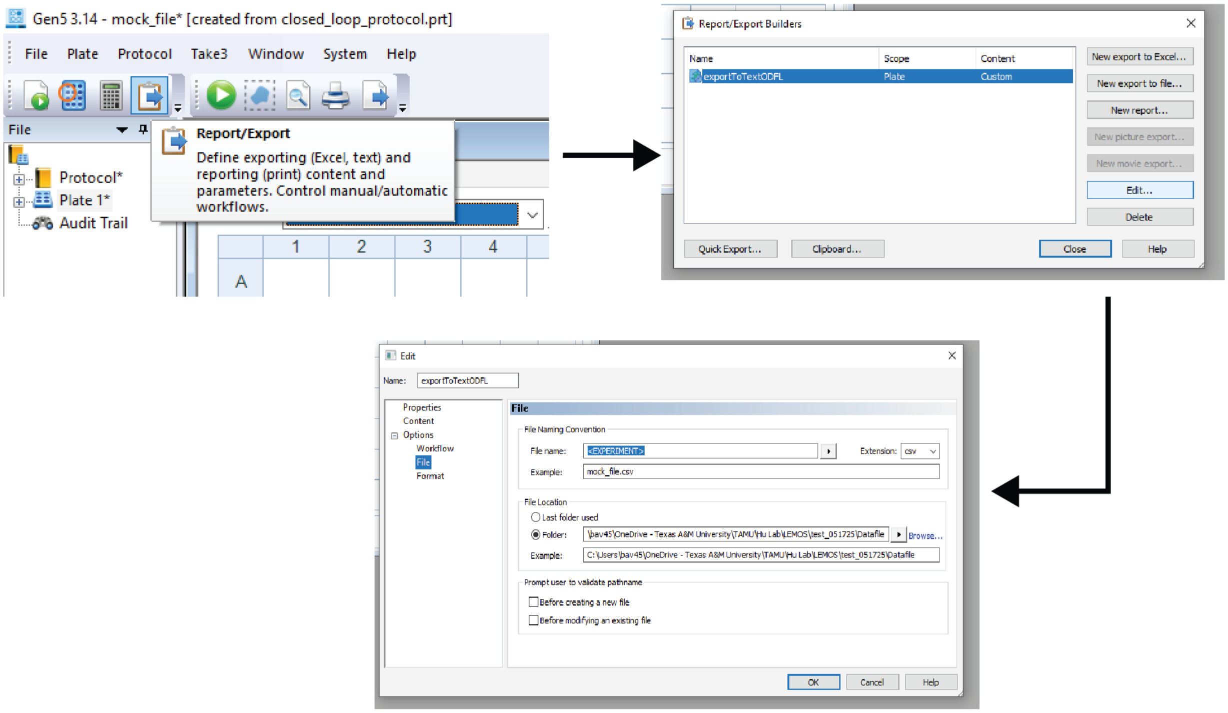

Fig. S2 Choosing the directory for experiment data export

d) Click on ‘Procedure’ to change the incubation temperature if required (Fig. S3).

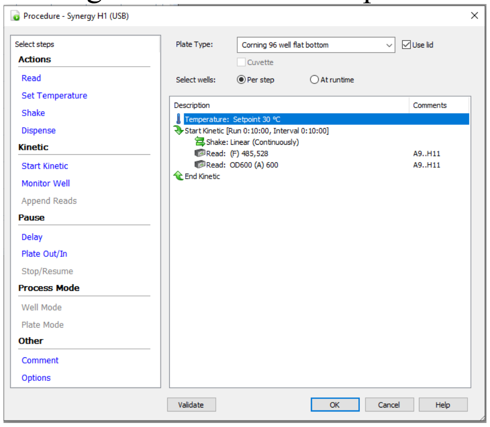

Fig. S3 Changing the incubation temperature

e) Click on ‘Run’, wait for the microplate reader to reach the desired incubation temperature, do not hit override or that will produce an error in the python script (Fig. S4).

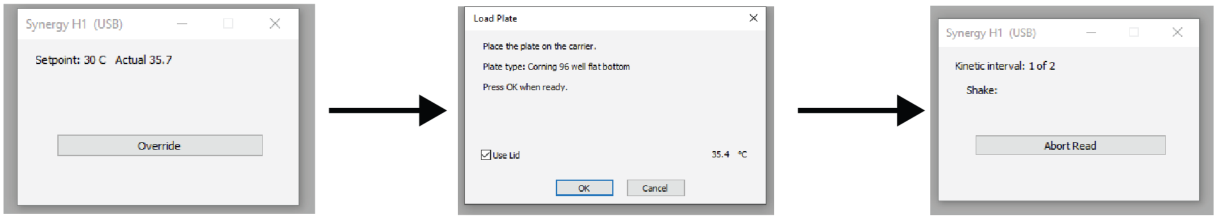

Fig. S4 Running the LEMOS experiment protocol

f) Once the microplate reader is at the desired temperature click ‘OK’.

g) Once the study starts running, hit ‘Abort Read’. This step has to be done only once in the beginning for the Gen5 to prompt the ‘Continue Reading Plate 1’ whenever ‘Run’ is selected by the python script (Fig. S5).

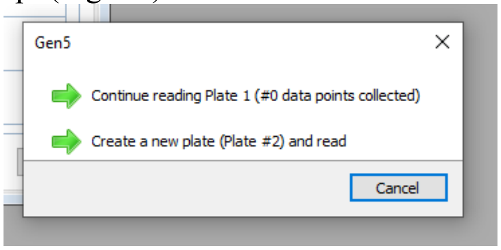

Fig. S5 Prompt to continue the reading in automated way

Python operation:

h) Select the folder where the data from the Gen5 software will be exported. This folder should be updated in the python script accordingly as described earlier (Fig. S6).

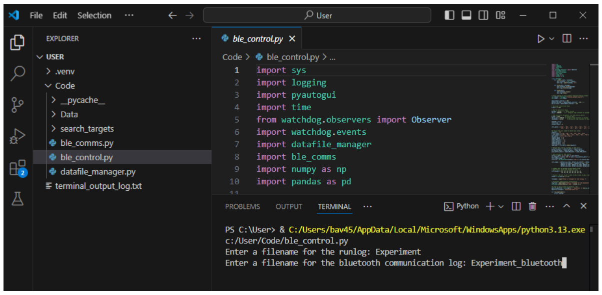

Fig. S6 Setting up the right directory location in Python script

i) Launch the python script

Growth Dynamics of *E.coli* under Different Illumination Conditions

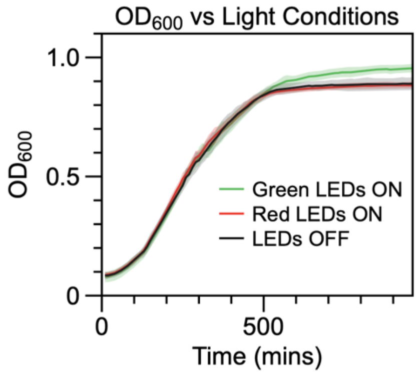

Fig. S7 Growth dynamics of E. coli under constant green, constant red and dark conditions.

Temperature effects of LED illumination in LEMOS wells

To assess potential LED-induced heating, we measured the temperature of water in the wells every 10 minutes over 70 minutes using an Extech thermocouple with a K-type probe (Fig. S8). The LEDs were operated at the same intensity used in the experiments reported in the manuscript.

First, we performed these measurements with the device stationary on the bench at ambient temperature. For both LED ON and LED OFF conditions, three technical replicates were collected to assess measurement variability. The temperature profile in illuminated and dark wells closely overlapped (Fig. S8A), indicating that LED illumination does not substantially increase the temperature in the wells.

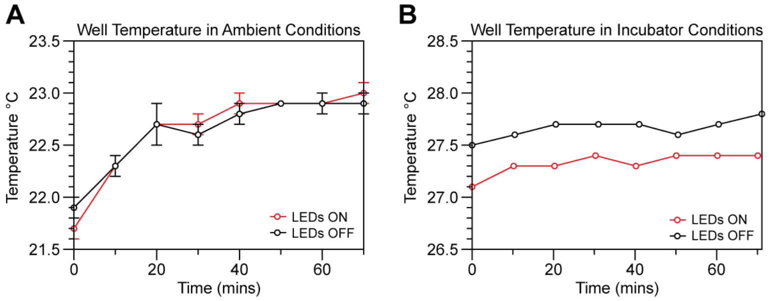

Fig. S8 Temperature variation in LEMOS microwells under varying conditions. (A) Temperature variation under ambient conditions. Wells equilibrate with the ambient conditions after ≈30 minutes. The small error bars further indicate that the measurements were consistent and that the variation was within the reported thermocouple accuracy of ±2.2 °C. (B) Temperature variation under incubator conditions. The difference between the two profiles is within the measurement variability observed in (A).

Next, to test the heating effect of LEDs in regulated conditions as in the experiments, we repeated the experiment in a 30 °C incubator. Before measurement, the LEMOS device was equilibrated in the incubator for 3 h. We limited this experiment to one replicate to minimize temperature drift during measurement, because each temperature reading required briefly removing the plate to room temperature and waiting 30 to 40 seconds for the thermocouple signal to stabilize. Under these conditions, the temperature in LED ON wells did not exceed that in LED OFF wells (Fig. S8B), again indicating that LED illumination does not increase the temperature in the wells.

Because these measurements were performed with the plate kept stationary, they likely overestimate local heating during actual experiments. In practice, the plates are continuously shaken, which should enhance heat transfer within the wells and help maintain culture temperatures closer to the 30 °C incubator set point.

Growth Curves at Different Duty cycles

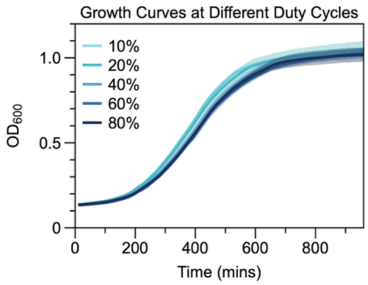

Fig. S9 Growth curves obtained from the duty cycle variation experiment described in Fig. 2E in the manuscript.

Duty Cycle Dynamics of P-Controller

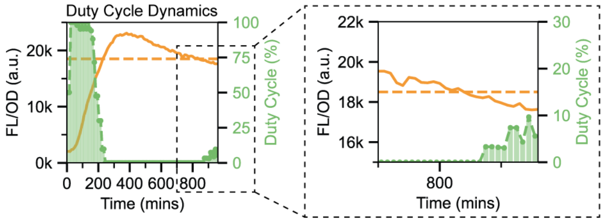

Fig. S10 Duty cycle dynamics of one SP2 replicate under P-control regulation. The zoomed in figure in the dotted lines show that the duty cycle increased when the signal dipped below the setpoint after ≈850 min.

Mono-Scale Model

To investigate the consistent overshoot observed under P-control regulation, we developed a deterministic kinetic model to capture the system's dynamic behavior. We used a Chemical Reaction Network (CRN) to represent the signal sensing and transduction dynamics of the two-component optogenetic system, and a set of ordinary differential equations (ODEs) to model the downstream gene expression process.

The kinetics of the sensing component is described with the following CRNs:

$$CcaS \underset{k_{rvrs}}{\overset{k_{fwd}}{\rightleftarrows}} CcaS_P \quad S1$$

$$CcaS_P + CcaR \rightleftarrows CcaS + CcaR_P \quad S2$$

$$CcaR_P + CcaR_P \rightleftarrows Ac \quad S3$$

$$k_{fwd} = k_{green} + k_{fb} \quad S4$$

$$k_{rvrs} = k_{red} + k_{rb} \quad S5$$

These reactions are shown in the schematic Fig. 2a. The sensing component of the optogenetic system begins with the transmembrane sensor $CcaS$, which becomes phosphorylated ($CcaS_P$) when exposed to green light. $CcaS_P$ then transfers the phosphate group to the response regulator $CcaR$, producing phosphorylated $CcaR$ ($CcaR_P$), which dimerizes to form the active transcription factor complex Ac. This activating complex initiates transcription of the sfGFP gene. To model light-dependent phosphorylation of CcaS, we defined the forward rate constant in equation (S1) as the sum of a small basal phosphorylation rate (k_{fb}) and the green-light–dependent rate (k_{green}) (equation (S4)). Under green light, phosphorylation occurs at the full rate $k_{green} + k_{fb}$, whereas in its absence (red light or darkness), only the basal rate applies (k_{fb}). Similarly, the $CcaR_P$dephosphorylation rate constant was defined as the sum of a basal dephosphorylation rate k_{rb} and the red-light–dependent rate (k_{red}), as described in equation (S5). Under red light, dephosphorylation occurs at the full rate $k_{red} + k_{rb}$, while in its absence (green light or darkness), only the basal rate (k_{rb}) is applied. The downstream processes of transcription, translation, and protein maturation are captured by the following system of ODEs described in equations S11-S13.

Here, Kc is the dissociation constant for the activating complex Ac activating the P_{cpcG2} promoter. The gene expression dynamics were modeled using the standard gene expression modeling framework[1], containing three main species including mRNA (M), unfolded sfGFP (P), and folded sfGFP (P_m). In this system, β and k_{tl} represent the transcription and translation rates, respectively; d_m denotes the mRNA degradation rate; d_p accounts for the protein dilution; and k_{fold} represents the protein maturation rate.

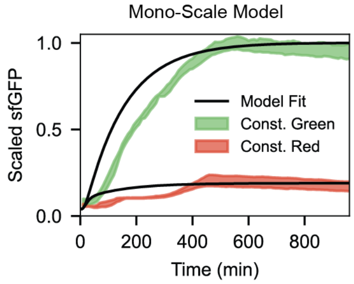

Fig. S11 Mono-scale model simulation of scaled sfGFP under constant green light activation and constant red-light repression, overlaid with experimental data (N=4, n=2); black solid line indicates the model fit. N denotes the number of technical replicates; n denotes for the number of biological replicates.

Model ODEs

$$\frac{dCcaS}{dt} = -(k_{green} + k_{fb}) \cdot CcaS + (k_{red} + k_{rb}) \cdot CcaS_P + k_{R_b} \cdot CcaS_p \cdot CcaR - k_{R_u} \cdot CcaS \cdot CcaR_P \quad S6$$

$$\frac{dCcaS_p}{dt} = (k_{green} + k_{fb}) \cdot CcaS - (k_{red} + k_{rb}) \cdot CcaS_P - k_{R_b} \cdot CcaS_p \cdot CcaR + k_{R_u} \cdot CcaS \cdot CcaR_P \quad S7$$

$$\frac{dCcaR}{dt} = -k_{R_b} \cdot CcaS_p \cdot CcaR + k_{R_u} \cdot CcaS \cdot CcaR_P \quad S8$$

$$\frac{dCcaR_p}{dt} = k_{R_b} \cdot CcaS_p \cdot CcaR - k_{R_u} \cdot CcaS \cdot CcaR_P - k_{Rp_b} \cdot CcaR_p^2 + k_{Rp_u} \cdot Ac \quad S9$$

$$\frac{dAc}{dt} = k_{Rp_b} \cdot CcaR_p^2 - k_{Rp_u} \cdot Ac \quad S10$$

$$\frac{dM}{dt} = \beta_m \cdot \left(\frac{Ac}{K_c + Ac} + l_0\right) - d_m \cdot M \quad S11$$

$$\frac{dP}{dt} = k_{tl} \cdot M - (d_p + k_{fold}) \cdot P \quad S12$$

$$\frac{dP_m}{dt} = k_{fold} \cdot P - d_p \cdot P_m \quad S13$$

Model species

Species explicitly modeled in the process:

Table S2 Growth independent model species

Species	Description
M	mRNA coding for sfGFP
P	Unfolded sfGFP
P_m	Folded sfGFP
$CcaS$	CcaS (membrane protein)
$CcaS_P$	Phosphorylated CcaS
$CcaR$	CcaR (response regulator protein)
$CcaR_P$	Phosphorylated CcaR
Ac	Transcription activation complex

Model parameters

Table S3 Growth independent model parameters

Parameter	Description	Unit	Value
β_m	Transcription rate per plasmid	$nM \cdot min^{-1}$	1
l_0	Leak coefficient of promoter	N/A	1.5e-1
K_c	Dissociation constant of Ac binding to promoter	nM	4
d_m	mRNA degradation rate constant	min^{-1}	1e-1
k_{tl}	Translation elongation rate	min^{-1}	1
d_p	Protein degradation rate	min^{-1}	7e-3
k_{fold}	sfGFP maturation rate	min^{-1}	1e-1
k_{green}	Phosphorylation rate of CcaS under green light	min^{-1}	1
k_{fb}	Basal phosphorylation rate of CcaS	min^{-1}	1e-1
k_{red}	Dephosphorylation rate of CcaS under red light	min^{-1}	8e-1
k_{rb}	Basal dephosphorylation rate of CcaS under red light exposure	min^{-1}	4e-1
k_{R_b}	Phosphorylation rate of CcaR by $CcaS_p$	$nM \cdot min^{-1}$	5e-2
k_{R_u}	Dephosphorylation rate of $CcaR_p$	$nM \cdot min^{-1}$	2.5e1
$k_{R_{pb}}$	Forward dimerization rate of $CcaR_p$	$nM \cdot min^{-1}$	100
k_{Rp_u}	Reverse dimerization rate of $CcaR_p$	min^{-1}	0.5

GEAGS model

To incorporate both growth and gene expression dynamics, we applied our previously established dual-scale Gene Expression Across Growth Stages (GEAGS) model framework[2]. In this model, equations (S18-S21) replace the earlier gene expression equations (S11–S13), while the phosphorylation reactions equations (S1–S3) remain unchanged. The dual-scale model combines ordinary differential equations (ODEs equations (S18-S21)) and chemical reaction network (CRN) equations (S14–S15) to capture gene expression dynamics, with an additional growth equation (equation (S22)) representing cell population dynamics.

In the growth dynamics equation (S22), C represents the cell count and C_{max} is the carrying capacity, defined by the nutrient availability and volume of the batch culture. The rate modifying functions (RMFs), α, δ and γ, capture how gene expression dynamics change as cells transition through different stages of growth. These RMFs are defined in terms of the normalized cell density $f = \frac{C}{C_{max}}$, which reflects the proximity of the population to its carrying capacity C_{max}. The first RMF, $\alpha = 1 - f$, represents the effective rate of cell division as biomass accumulates in the batch culture. In the model, α modulates the dilution rates of mRNA as well as both folded and unfolded sfGFP. The 2nd RMF, $\delta = \frac{f^n}{1+f^n}$, where n is the Hill coefficient, influences the steepness of the function δ. In the model, δ modulates the degradation of both folded and unfolded sfGFP, capturing the upregulation of proteolysis as cells enter stationary phase. The 3rd RMF $\gamma = \left(f \cdot (1-f)\right)^m$, reflects the molecular process efficiency across growth phases, peaking during mid-log when growth is most rapid. In the model, γ modulates the effective transcription rate, sfGFP maturation rate and the coarse-grained translation resource (R) availability. Additional parameters in the growth dependent model include d_{dil}, the dilution rate due to cell growth, and $k_{tli_b}$and k_{tli_u}, the binding and unbinding rates of the coarse-grained translation initiation complex C_{tic}, respectively. The dynamics of R and C_{tic} are captured by the following equations:

$$R_{free} + M \underset{k_{tli_u}}{\overset{k_{tli_b}}{\rightleftarrows}} C_{TIC} \qquad S14$$

$$C_{TIC} \xrightarrow{k_{tl}} P + M + R_{free} \qquad S15$$

$$R_{total} = R_{max} \cdot \gamma \qquad S16$$

$$R_{free} = R_{total} - C_{TIC} \qquad S17$$

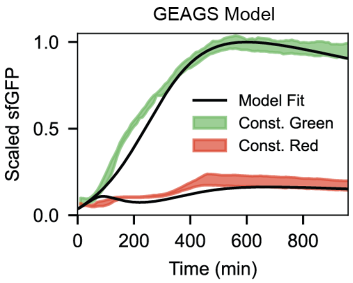

Fig. S12 Dual-scale GEAGS (Gene Expression Across Growth Stages) model simulation overlaid with the same constant-input data in Fig. S11.

GEAGS Model ODEs

$$\frac{dM}{dt} = \boldsymbol{\beta_m} \cdot \left(\frac{Ac}{K_c + Ac} + l_0\right) - (\boldsymbol{d_m} + \boldsymbol{d_{dil}}) \cdot M - k_{tli_b} \cdot \boldsymbol{R_{free}} \cdot M + k_{tli_u} \cdot C_{tic} + k_{tl} \cdot C_{tic} \quad S18$$

$$\frac{dC_{tic}}{dt} = k_{tli_b} \cdot \boldsymbol{R_{free}} \cdot M - k_{tli_u} \cdot C_{tic} - k_{tl} \cdot C_{tic} \quad S19$$

$$\frac{dP}{dt} = k_{tl} \cdot C_{tic} - (\boldsymbol{d_p} + \boldsymbol{d_{dil}}) \cdot P - \boldsymbol{k_{fold}} \cdot P \quad S20$$

$$\frac{dP_m}{dt} = \boldsymbol{k_{fold}} \cdot P - (\boldsymbol{d_p} + \boldsymbol{d_{dil}}) \cdot P_m \quad S21$$

$$\frac{dC}{dt} = k_{gr} \cdot C \cdot \left(1 - \frac{C}{C_{max}}\right) \quad S22$$

Growth dependent rates:

Applying RMFs to growth-dependent rates:

$$\boldsymbol{\beta_m} = \beta_m \cdot \gamma \quad S23$$

$$\boldsymbol{k_{fold}} = k_{fold} \cdot (\gamma + b_{fold}) \quad S24$$

$$\boldsymbol{d_m} = d_m \cdot \alpha \quad S25$$

$$\boldsymbol{d_p} = d_p \cdot \delta \quad S26$$

$$\boldsymbol{d_{dil}} = d_{dil} \cdot \alpha \quad S27$$

Conservation equations for growth dependent translation resource (R):

$$R_{total} = R_{max} \cdot \gamma \quad S28$$

$$R_{free} = R_{total} - C_{tic} \quad S29$$

Model species

Table S4 GEAGS model species

Species	Description
M	mRNA coding for sfGFP
P	Unfolded sfGFP
P_m	Folded sfGFP

$CcaS$	CcaS (membrane protein)
$CcaS_P$	Phosphorylated CcaS
$CcaR$	CcaR (response regulator protein)
$CcaR_P$	Phosphorylated CcaR
Ac	Transcription activation complex
C_{tic}	Translation initiation complex
R	Coarse-grained translation resource
C	Cell population

Model parameters

Table S5 GEAGS model parameters

Parameter	Description	Unit	Estimate
β_m	Transcription rate per plasmid	$nM \cdot min^{-1}$	2.8e1
l_0	Leak coefficient of promoter	N/A	1e-5
K_c	Dissociation constant of Ac binding to promoter	nM	4.5e1
d_m	mRNA degradation rate constant	min^{-1}	2.7e-1
k_{tli_b}	C_{tic} formation rate	$nM^{-1} \cdot min^{-1}$	4e1
k_{tli_u}	C_{tic} dissociation rate	min^{-1}	1e1
k_{tl}	Translation elongation rate	min^{-1}	2
d_p	Protein degradation rate	min^{-1}	8e-4
k_{fold}	sfGFP maturation rate	min^{-1}	3e-1
b_{fold}	Basal coefficient for k_{fold}	N/A	1
k_{green}	Phosphorylation rate of CcaS under green light	min^{-1}	8e-1
k_{fb}	Basal phosphorylation rate of CcaS	min^{-1}	3e-2
k_{red}	Dephosphorylation rate of CcaS under red light	min^{-1}	1.3
k_{rb}	Basal dephosphorylation rate of CcaS under red light exposure	min^{-1}	8e-1
k_{R_b}	Phosphorylation rate of CcaR by $CcaS_p$	$nM \cdot min^{-1}$	5e-2
k_{R_u}	Dephosphorylation rate of $CcaR_p$	$nM \cdot min^{-1}$	2.5e1
$k_{R_{pb}}$	Forward dimerization rate of $CcaR_p$	$nM \cdot min^{-1}$	5.5e1
k_{Rp_u}	Reverse dimerization rate of $CcaR_p$	min^{-1}	8e-2
R_{max}	Max. total R availability	nM	4
n	Exponent of γ	N/A	8.9e-1
n_{delta}	Hill coefficient of δ	N/A	5.5
C_0	Initial condition for cell population	$counts$	4.69e7
C_{max}	Max. cell population (holding capacity)	$counts$	7.14e8
k_{gr}	Logistic growth rate	min^{-1}	1.1e-2

Minimal GEAGS Model

Model ODEs

$$\frac{dM}{dt} = \boldsymbol{\beta_m} \cdot \left(\frac{Ac}{K_c + Ac} + l_0\right) - (\boldsymbol{d_m} + \boldsymbol{d_{dil}}) \cdot M \qquad S30$$

$$\frac{dP}{dt} = \boldsymbol{k_{tl}} \cdot M - (\boldsymbol{d_p} + \boldsymbol{d_{dil}}) \cdot P \qquad S31$$

$$\frac{dC}{dt} = k_{gr} \cdot C \cdot \left(1 - \frac{C}{C_{max}}\right) \qquad S32$$

Modified rate equations:

$$\boldsymbol{\beta_m} = \beta_m \cdot \gamma \qquad S33$$

$$\boldsymbol{k_{tl}} = k_{tl} \cdot \gamma \qquad S34$$

$$\boldsymbol{d_m} = d_m \cdot \alpha \qquad S35$$

$$\boldsymbol{d_p} = d_p \cdot \delta \qquad S36$$

$$\boldsymbol{d_{dil}} = d_{dil} \cdot \alpha \qquad S37$$

Model species

Table S6 Minimal model species

Species	Description
M	mRNA coding for sfGFP
P	sfGFP
C	Cell population

Model parameters

Table S7 Minimal model parameters

Parameter	Description	Unit	Estimate
β_m	Transcription rate per plasmid	$nM \cdot min^{-1}$	3
l_0	Leak coefficient of promoter	N/A	5e-3
K_c	Dissociation constant of Ac binding to promoter	nM	10
d_m	mRNA degradation rate constant	min^{-1}	2e-1
k_{tl}	Translation elongation rate	min^{-1}	1.1
d_p	Protein degradation rate	min^{-1}	1e-4
n	Exponent of γ	N/A	0.9
n_{delta}	Hill coefficient of δ	N/A	5.5
C_0	Initial condition for cell population	$counts$	4.69e7
C_{max}	Max. cell population (holding capacity)	$counts$	8.37e8
k_{gr}	Logistic growth rate	min^{-1}	1.2e-2

Assume steady state values of Ac for each light input:

Under green light: $Ac = 80\ nM$
Under red light: $Ac = 1\ nM$
Under no light (dark): $Ac = 3\ nM$

Parameter Estimates

This section briefly summarizes the parameter estimates and the rationale used to obtain the initial values. Starting from these initial guesses, we then manually tuned the model parameters to reproduce the dynamics accurately.

The light-signaling and phosphorylation parameters are chosen so that the TCS module operates on a fast time scale while staying within experimentally reasonable rate ranges for protein–protein interactions and phosphorylation. Effective phosphorylation rates on the order of 0.8–2 min^{-1} ensure that switching between S and Sp occurs 0.5–1 min, which matches typical sensor kinase response times[3]. Binding/unbinding parameters for $CcaS_P - CcaR$ and $CcaR_P - CcaR_P$ /Ac are kept in the same order-of-magnitude window as diffusion-limited protein–protein association and dissociation in the cytoplasm[4].

To obtain the transcription rate, we started from an estimate of 3.72 kb/min, which corresponds to ≈3.72 transcripts per minute[5]. Accounting for a medium plasmid copy number and the multiplication with the hill function, we estimate the effective transcription rate to be of the order 10-50 mRNA/min. Translation elongation rates in E. coli are typically 9–16 amino acids per second[6], i.e. an average 300-aa protein is synthesized in roughly 15–80 s, corresponding to an effective elongation rate constant of order 1 – 4 min^{-1}. The binding and unbinding rates of the translation resource complex were set to 40 $\mathrm{nM}^{-1}\mathrm{min}^{-1}$ and 12 $\mathrm{nM}^{-1}\mathrm{min}^{-1}$, corresponding to $k_{\mathrm{on}} \approx 6.7 \times 10^{8}\ \mathrm{M}^{-1}\mathrm{s}^{-1}$, $k_{\mathrm{off}} \approx 0.2\ \mathrm{s}^{-1}$, and an effective dissociation constant $K_D \approx 0.3$ nM. These values lie below the diffusion limit for macromolecular association and yield complex lifetimes of a few seconds, which is consistent with measured ribosome–mRNA interaction times in bacteria[4]. The protein maturation rate was estimated to be 0.2 min^{-1}based on the super-folder green fluorescent protein, which was estimated to mature within 6 minutes in *E. coli*[7].

We obtained the estimate of the mRNA degradation as 0.25 min^{-1} from the known degradation times of 2 – 5 minutes[1] and protein degradation as 8e-4 min^{-1} from known degradation half-lives of >24 hrs[8].

Model Performance under Gain Perturbation

To assess the impact of gain variations on model performance, we first selected 50 random gain values from a uniform distribution ±15% around the nominal values, for all the three controllers. For each random gain, we simulated the controller and measured the settling time and overshoot error. The results from this simulation, shown in Fig. S13, are consistent with the behavior observed in Figures 5F and 5H in the manuscript. This suggests that the model is robust to gain changes, demonstrating stable performance under parameter uncertainty.

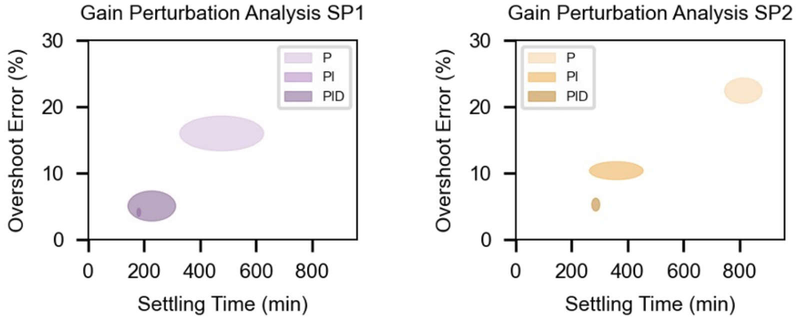

Fig. S13 Performance analysis of the controller models under gain perturbations.

<u>References</u>

(1) Alon, U. *An introduction to systems biology: design principles of biological circuits*; Chapman and Hall/CRC, 2019.

(2) Namboothiri, H. R.; Pandey, A.; Hu, C. Y. Resolving emergent transient oscillations in gene circuits with a growth-coupled model. *Sci Adv* **2026**, *12* (6), eadz2310. DOI: 10.1126/sciadv.adz2310.

(3) Gao, R.; Stock, A. M. Quantitative Kinetic Analyses of Shutting Off a Two-Component System. *mBio* **2017**, *8* (3). DOI: 10.1128/mBio.00412-17.

(4) Schlosshauer, M.; Baker, D. Realistic protein-protein association rates from a simple diffusional model neglecting long-range interactions, free energy barriers, and landscape ruggedness. *Protein Sci* **2004**, *13* (6), 1660-1669. DOI: 10.1110/ps.03517304.

(5) Proshkin, S.; Rahmouni, A. R.; Mironov, A.; Nudler, E. Cooperation between translating ribosomes and RNA polymerase in transcription elongation. *Science* **2010**, *328* (5977), 504-508. DOI: 10.1126/science.1184939.

(6) Sorensen, M. A.; Fehler, A. O.; Lo Svenningsen, S. Transfer RNA instability as a stress response in Escherichia coli: Rapid dynamics of the tRNA pool as a function of demand. *RNA Biol* **2018**, *15* (4-5), 586-593. DOI: 10.1080/15476286.2017.1391440.

(7) Megerle, J. A.; Fritz, G.; Gerland, U.; Jung, K.; Radler, J. O. Timing and dynamics of single cell gene expression in the arabinose utilization system. *Biophys J* **2008**, *95* (4), 2103-2115. DOI: 10.1529/biophysj.107.127191.

(8) Maurizi, M. R. Proteases and protein degradation in Escherichia coli. *Experientia* **1992**, *48* (2), 178-201. DOI: 10.1007/BF01923511.
